# A Simple Approach for Measuring Emission Patterns of Vapor Phase Mercury under Temperature-Controlled Conditions from Soil

**DOI:** 10.1100/2012/940413

**Published:** 2012-08-13

**Authors:** Ki-Hyun Kim, Hye-On Yoon, Myung-Chae Jung, Jong-Min Oh, Richard J. C. Brown

**Affiliations:** ^1^Department of Environment and Energy, Sejong University, Seoul 143-747, Republic of Korea; ^2^Korea Basic Science Institute, Seoul Center, Anamdong, Seoul 136-713, Republic of Korea; ^3^Department of Energy and Mineral Resources Engineering, Sejong University, Seoul 143-747, Republic of Korea; ^4^College of Environment and Applied Chemistry, Kyung Hee University, Yongin 449-701, Republic of Korea; ^5^Analytical Science Division, National Physical Laboratory, Hampton Road, Teddington TW11 0LW, UK

## Abstract

In an effort to study the possible effects of climate change on the behavior of atmospheric mercury (Hg), we built a temperature–controlled microchamber system to measure its emission from top soils. To this end, mercury vapour emission rates were investigated in the laboratory using top soil samples collected from an urban area. The emissions of Hg, when measured as a function of soil temperature (from ambient levels up to 70°C at increments of 10°C), showed a positive correlation with rising temperature. According to the continuous analyses of the Hg vapor given off by the identical soil samples, evasion rate diminished noticeably with increasing number of repetitions. The experimental results, if examined in terms of activation energy (Ea), showed highly contrasting patterns between the single and repetitive runs. Although the results of the former exhibited Ea values smaller than the vaporization energy of Hg (i.e., <14 Kcal mol^−1^), those of the latter increased systematically with increasing number of repetitions. As such, it is proposed that changes in the magnitude of Ea values can be used as a highly sensitive criterion to discriminate the important role of vaporization from other diverse (biotic/abiotic) processes occurring in the soil layer.

## 1. Introduction

 As one of the most toxic and bioconcentrated trace metals in the aquatic and terrestrial food chain, the environmental behavior and the associated atmospheric and terrestrial cycle of mercury (Hg) have become a growing theme in many fields of geosciences, in particular in atmospheric sciences [1]. Focused research efforts have yielded fruitful outcomes in estimating global and/or regional scale Hg budgets and in explaining the behavior of Hg in various media [2–5]. Despite the progresses achieved over recent years, it is still difficult to provide a concrete assessment of the relationships between the scale of natural and anthropogenic source processes. Confusion surrounding this issue is as a result of man-made sources showing significant variation, brought about by rapid changes in Hg control and abatement strategies. Moreover, it is also recognized that the relative sizes of the different natural (in particular terrestrial) sources are not yet firmly established. This is clearly important in order to account for their relative contributions to the global Hg budget [6].

The soil-to-air exchange of Hg is characterized by a reproducible diurnal trend correlated with changes in solar radiation, while also being affected by many other environmental variables (e.g., soil temperature, wind speed, vertical mixing, latent energy, precipitation, vegetation surface, etc.) [7–9]. All the processes leading to active transfer of Hg across an air-surface boundary may occur many times within the geochemical cycle via (re-) emission and (re-) deposition, and so forth. This becomes particularly important if the natural budgets of Hg are compared across its natural reservoirs; Hg in soil (1.2 × 10^6^ Mg) is estimated to exceed substantially its sum in the ocean (3.6 × 10^5^ Mg) and atmosphere (6 × 10^3^ Mg) [10]. As such, it is reasonable to infer that the role of Hg in soil will become even more prominent with the progress of climate change.

Although the emission of Hg from soil is dependent on many variables [11], we examined the fundamental properties of Hg exchange from soil in response to temperature rise in order to demonstrate the performance of our novel apparatus. Whilst this effect is well known (being examined previously (e.g., [12]), we have now designed a new and simple apparatus to examine the phenomenon on some different aspects. To analyze the interaction between Hg behavior and changing soil temperature, a novel impinger system modified for flux measurements (e.g., a microchamber system) was used to quantify its exchange rates, using soil samples collected in an urban area. The results of this analysis will be useful to assess the basic aspects of Hg exchange under the temperature-regulated conditions and may help predict the increased burden of Hg in the atmosphere as a result of average increases in ambient temperatures.

## 2. Materials and Methods

### 2.1. Sample Collection and Analysis

**Figure 1:**
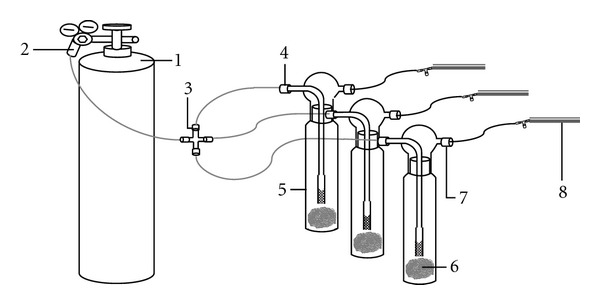
Illustration of the sampling apparatus for sediment flux analysis using an impinger vessel as a microflux chamber (1) ultrapure air tank; (2) ultrapure air flow regulator; (3) flow control/regulator towards the 3 impingers; (4) impinger inlet; (5) impinger bottle (6) sediment sample (50 g); (7) impinger outlet; and (8) adsorption tubes.

In order to obtain the Hg emission data from soil systems in relation to rising soil temperature, a simple experimental system was devised to allow the simultaneous collection of Hg samples from three impingers (as microchambers) in combination with a temperature controlling water bath (Figure 1). In order to improve the detection limits for the Hg released from soil samples, the total volume of air passing through the impinger was maintained at 120 L (4 hrs of sampling at a flow rate of 0.5 L min^−1^). In light of the volume of the impinger used in this research (0.5 L), one would expect a chamber turnover time of the order of one to two minutes with the flow being expected to be laminar through the impinger. Whilst chamber turnover will have an influence on Hg flux, we do not consider this parameter further in this study. It is because this turnover rate is fairly high and, in any case, this is a comparative laboratory study.

 As sampling duration is relatively long for each run (e.g., 4 hrs duration for the acquisition of a single datapoint at a given temperature), all of our analysis collected data from 3 samples at a time. For simple comparison, a total of 3 different soil samples were taken from 3 different spots (several meters apart from each other) in the front yard of Young Sil Building, Sejong University, Republic of Korea during January 2010. All of these soil samples represent the upper (top) soil layers (e.g., O, A, and B horizons; 0–30 cm). Care was taken to minimize the intermixing of soil materials using a hand spade. The basic soil properties determined for these 3 soil samples are given in Table 1 along with the concentrations of Hg within the soil. The results indicate that the Hg contents of the three soil samples are similar and all in the range 2.8 to 3.3 mg kg^−1^, rather elevated compared to background soil Hg concentrations (<0.2 mg kg^−1^). 

As shown in Figure 1, the system is built to simultaneously undertake flux measurements in each of the 3 impingers, under the same temperature conditions. Our triplicate sampling system was hence used to simultaneously measure the Hg emission rates for three different soil sample masses: 0 (blank), 50, and 100 g. This chamber system thus provided blank levels for the correction of analytical values derived at two soil quantities (50 and 100 g) as required. To initiate each experiment, the inlet and outlet of each impinger are connected to the purging system (cylinder filled with ultrapure air) and the sample collection system (Au amalgam tube), respectively (Figure 1). The ultrapure air from the cylinder is then released and brought into each of the 3 impingers at a fixed flow rate of 500 mL min^−1^ for 4 hours, to make a total sample volume of 120 L at each temperature.

 The exchange rates of Hg for a given soil sample were quantified continuously by raising the soil temperature from room temperature up to 70°C in 10°C intervals. The flux data taken from the blank samples averaged 0.11 ± 0.08 ng m^−3^ (*n* = 27) which corresponds to 2 or 0.7% of Hg levels determined from 50 and 100 g soils, respectively. These blank values for each experiment were used for the adjustment of the Hg values measured for soil samples with 50 and 100 g. As the temperature for each experiment was held constant for 4 hrs, it typically took 3 to 4 days to acquire the data over a full temperature range for a given soil sample. Once a new set of experiments started, impingers containing soil samples were left in the laboratory with their open ends sealed by Mylar film. All the samples were kept at room temperature under the standard fluorescent lamp in the laboratory throughout the study period. A fluorescent tube is known to be a more diffuse and physically larger light source than an incandescent lamp. All experiments were hence conducted without the complete removal of light intensity. Although light has been identified to be one of the most sensitive variables controlling Hg emission behavior, this study was mainly intended to measure the effect of soil temperature change on its emission rate without the control of light conditions—which were constant throughout.

The analysis of the Hg collected in the adsorption tubes was performed using thermal desorption (350°C) and detection at a wavelength of 253.7 nm with a nondispersive double beam and flameless atomic absorption mercury analyzer (WA-4, Nippon Instrument Co., Japan). The specification and results of analytical performance for the comparable instrumental setup can be found in our previous studies, described elsewhere [13–15]. The method detection limit (DL) of the system was slightly variable from day-to-day but did not exceed 10 pg of Hg mass; this DL is moderately higher than those reported by the similar instrumental systems in our previous study (e.g., [13])—but much lower than any of the concentration expected in this study. DL values were obtained as 3.14 times the standard deviation values of 7 repeat runs at intensities just distinguishable from system blanks.

### 2.2. Derivation of Hg Flux

The emission rate of Hg was quantified by inputting the measured Hg concentration determined from each experiment into the following formula:

(1) $$\begin{array}{c} F=\;C\;\left(L\;+\frac{Q}{A}\;\right), \end{array}$$

where *F* = Hg flux in dimensions of mass per area per time (ng m^−2 ^min^−1^); *C* = Hg concentration exiting the chamber (ng m^−3^); *Q* = flow rate passing through the chamber (m^3^ min^−1^); and *A* = inner surface area of the chamber (m^2^).

By assuming that the loss term is almost negligible (because we assume—and have evidence—that the chamber walls have been fully conditioned with Hg during setup and validation such that there is no longer any net transfer of Hg from gaseous to adsorbed phases), the flux values of Hg can be approximated by multiplying Hg concentration exiting the chamber by the ratio *Q*/*A*, that is, *F* = *QC*/*A*. 

## 3. Results and Discussion

### 3.1. The Pattern of the Hg Emission Data in Relation to Soil Quantity Criterion

 In the initial stage of our experiments, in order to evaluate the emission of vapor phase Hg from different soil samples, we measured Hg fluxes from 3 independent soil samples labeled as S1, S2, and S3. In the second stage of our experiment, the soil emission rate for one of those three soil samples (S3) was measured consecutively three times. Each of these experimental runs covered the full temperature range (from room temperature to 70°C in 10 degree steps) and was compared to examine the trend over repetitive runs. Although temperature was a key variable in our investigations, we also briefly examined the effect of sample quantity on observed emission rates. As a result of this objective, our experiments were conducted using 2 different soil quantities: 50 and 100 g. To resolve any differences in the emissions from these two soil weights, these results were treated independently and are summarized in Tables 2 and 3, respectively. For completeness, the Hg concentrations used for the derivation of the flux values are also presented.

**Figure 2:**
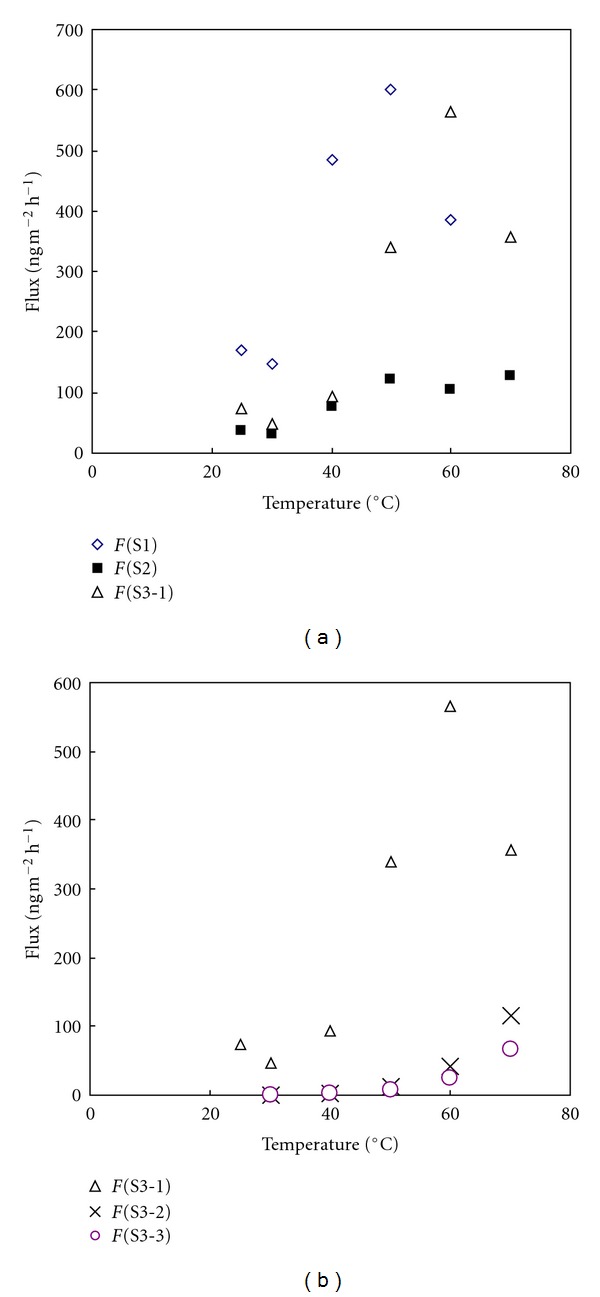
Relationship between temperature and Hg flux (ng m^−2^ h^−1^) from 100 g soil samples: (a) Hg flux values derived from the initial run of three samples (upper) and (b) Hg flux values derived from the repetitive runs of S3 sample.

The volume of the impinger chamber system used in this study is not large enough to contain more than 100 g of soils. As such, the data presented in this preliminary study is not able to examine the effect of soil sample quantities other than at those two values (50 and 100 g)—especially weights in excess of 100 g. Nonetheless, comparison of these data indicates that the emissions of Hg from soil under these experimental conditions may be affected greatly by the weight of soil placed in the impinger. Most importantly, it is apparent that the experimental data obtained using the 100 g soil samples tend to exhibit fairly systematic trends of increasing emissions as temperature increased (Figure 2) similarly to the original observations of this type [12]. This is however not the case for the 50 g samples, which did not show any Hg emissions up to 50°C in the initial experiments. In addition, Hg flux data from the different 50 g samples showed highly contrasting patterns; although the maximum emission value of the S1 sample was seen at 50°C, those of S3 occurred at the lowest temperature of 25°C. Moreover, in our repeated analysis of 50 g samples, only small quantities of Hg were emitted consistently after 60°C. As such, the results derived using 50 g soil samples suggest that the emission of Hg from this quantity of soil is less likely to exhibit and maintain a systematic trend. As seen in Table 2, the results from all three 50 g samples confirm irregular patterns of Hg emission with increasing temperature. The effect observed with sample mass may be a function of the exposed surface area (which is constant for each mass—and constrained by the cross-sectional area of the impinger) to volume (which changes for each mass) ratio exhibited by each sample, although this hypothesis needs further testing.

### 3.2. Effect of Soil Temperature Rise on Hg Emission

 As plotted in Figure 2(a), the trend of increasing Hg emission with increases in soil temperature from 100 g soil samples is distinctive and systematic. The results presented in Table 3 indicate that Hg emissions gradually increased with increasing temperature without any noticeable exceptions. As the Hg emission trends from the 50 g soil samples were rather complicated to interpret, we focused mainly on the emission data from the 100 g soil samples to evaluate the relationship between temperature and Hg emissions. The results of the initial runs from the 3 soil samples (S1, S2, and S3) indicated consistently that the Hg emission rates increased almost exponentially with temperature, peaking in the range of 50 to 70°C. However, the magnitude of the emissions from the 3 samples differs moderately, for example, the maximum emission value of 126 ng m^−2^ h^−1^ for S2 in contrast to the maximum for the other two samples of near to 600 ng m^−2^ h^−1^ (Figure 2(a)). In addition, the results of 3 continuous runs of the S3 sample (S3-1, S3-2, and S3-3) indicate that the Hg can be released continuously by forced extraction (e.g., via repetitive heating). However, it was very clear that the magnitude of Hg emission diminished systematically with increasing numbers of repeated heating cycles (Figure 2(b)). If the emission values at 70°C are compared, the values measured were 357 (first heating cycle), 116 (second heating cycle), and 67 ng m^−2^ h^−1^ (third heating cycle). Nonetheless, the results from these repeated runs suggest that soil may act as a significant source of Hg to the atmosphere particularly as a result of changes in the surrounding climate. 

**Figure 3:**
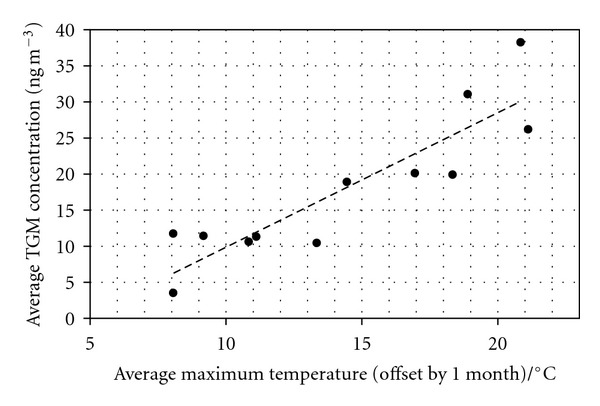
The average maximum monthly temperature (offset by one month to allow for the lag in the ground warming and cooling) against the average monthly total gaseous mercury concentration recorded at the UK Heavy Metals Monitoring Air Quality Network station at Runcorn during 2010. The best linear fit to the data is shown (*R* ^2^ = 0.8) as the dotted line.

In recent years, the important role of terrestrial source processes in Hg geochemistry has been highlighted because of its close linkage with the global warming trend. Because of its unique vaporization properties, the cycling of Hg is expected to share some common features with that observed for greenhouse gas pollutants [16]. The increasing evasion of Hg with rising temperature has in fact been demonstrated from both water and soil layers [17]. Indeed Figure 3 shows data from the UK Heavy Metals Monitoring Network Air Quality Network station at Runcorn [18], where historical industrial processes have led to contamination of the soil by mercury [19]. In such case, it can be clearly seen that the mercury vapor levels measured in air correlate well (*R* ^2^ = 0.8) with the average temperature at the monitoring sites (offset by one month to allow for the lag in the ground warming and cooling). That the correlation is worse when the temperature is not offset by one month (*R* ^2^ = 0.5) is a good indication that temperature is a major factor in causing more mercury evasion from soil and therefore increasing the measured total gaseous mercury (TGM) concentration in surrounding air, and not simply other factors such as light and ozone concentration which would have a more temporally direct effect. 

 To learn more about the factors regulating Hg emissions from soil, we examined our experimental results in relation to activation energy (*E*_*a*_). As documented by many previous studies, mercury fluxes over soils (and to a lesser extent ambient concentrations) generally exhibit a strong exponential relationship with changing soil temperature [12, 20, 21]. This temperature dependence of Hg emissions has often been accounted for by an interactive relationship between the physicochemical properties of elemental mercury (high vapor pressure and low water solubility) and biotic/abiotic processes occurring in the soil layer. As the mechanism of Hg emission from soil is particularly sensitive to temperature rises, its potential can be expressed in terms of activation energy (*E*_*a*_) with the aid of the Arrhenius equation [6, 22]. *E*_*a*_ values have thus often been derived to estimate the partitioning of Hg evasion from soil between (a) physical vaporization (due to its volatility) and (b) other soil processes with more biological/chemical nature (e.g., photochemical reduction (abiotic) or biological mediation (biotic)).

Previous estimates of activation energy showed that the values measured from various environmental surfaces can fall in a relatively wide range: 17.3 ± 7.7 in background forest [23], 20.53 over pasture fields [17], 25.8 ± 2.6 in contaminated soils [6], 28.0 ± 5.7 in mercuriferous volcanic soils [24], and 29.6 ± 1.0 Kcal mol^−1^ in lake surfaces in Sweden [22]. If this type of approach is extended to encompass a variety of environmental substances (e.g., lake sand, coated glass, and organic material) and between light and dark conditions, a broad range of *E*_*a*_ values are found from 5.2 to 152 Kcal mol^−1^ [20]. However, in light of the fact that the enthalpy of Hg^o^ vaporization is ~14 Kcal mol^−1^, the major driving force of Hg emission in each case may be estimated with respect to this reference *E*_*a*_ value. As such, our experimental *E*_*a*_ results were derived based on two-types of approaches (either the initial runs of each of 3 samples or repeated runs from a single soil sample (S3)), they can also be evaluated against such criterion.

**Figure 4:**
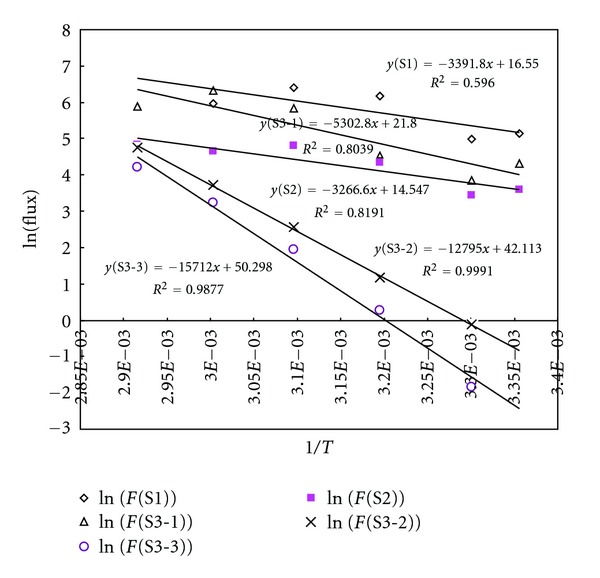
Relationship between 1/*T* (abs) and ln(Hg flux) to derive activation energy (*E*_*a*_).

Fitting the data obtained to the Arrhenius equation, the *E*_*a*_ values for the single run (S1, S2, and S3) samples were computed as 6.74, 6.49, and 10.5 Kcal mol^−1^ (Figure 4). These data are far lower than the enthalpy of vaporization for Hg and of those determined in previous studies. However, the results from the repeated runs exhibit a contrasting trend; their *E*_*a*_ values increase systematically with the number of repetitions such that the values obtained were 10.5 (S3—repeat 1), 25.4 (S3—repeat 2), and 31.2 Kcal mol^−1^ (S3—repeat 3). It is thus suggested that the emissions of Hg from single (or first) runs can be dominated directly by vaporization, whereas emissions observed from continuous runs are to be driven directly by the control of abiotic parameters. Once the majority of Hg emission is accounted for by vaporization, the effect of other processes becomes more prominent in regulating subsequent emissions.

 As the results of our study show, emissions of Hg from continuous runs require an extra source of energy other than vaporization. Gustin et al. [20] explained the light-induced photochemical reduction of reactive divalent Hg to elemental Hg as the important sources of soil-derived Hg. These authors observed that the magnitude of light-enhanced emissions for natural substrates were 1.5 to 116 times larger than that under the dark conditions. In addition, based on the findings of the relative enhancement of *E*_*a*_ during daytime, they postulated that the release of Hg from such substrates may increase due to phytoreduction when exposure to sunlight. Likewise, Choi and Holsen [25] observed that the Hg emissions were very sensitive to UV-B exposure (302 nm), while there were no such effects with UV-A (365 nm). Based on this finding, they suggested that the role of abiotic parameters is more prominent in controlling Hg emissions than biotic ones.

## 4. Conclusions

The present study was undertaken to investigate the factors involved in the emissions of Hg from soil to atmosphere and demonstrate the use of a novel apparatus to measure this. In an effort to elucidate the fundamental features of this process, we measured Hg emission fluxes from soil samples using a novel microchamber system across varying soil temperatures from room temperature up to 70°C. The emission rates of Hg were initially measured once from each of the three soil samples, and then one of these samples was selected and measured repeatedly (i.e., up to 3 consecutive runs) to determine the forced emission patterns with increased soil temperature.

The results of our laboratory study consistently indicate that there is an exponential increase in Hg emissions with rising temperature. In addition, the emission rates of Hg, measured repetitively using the same soil samples, were subject to a stepwise reduction with each subsequent heating cycle. The temperature dependence of Hg emissions, if evaluated in terms of activation energy (*E*_*a*_), complies well with general expectations. The computed *E*_*a*_ values indicate that the emission of Hg from soil is initially controlled by vaporization, while the subsequent reemission is driven by the biotic/abiotic processes occurring in the soil layer. It is thus reasonable to infer that the mercury liberated during the second and third repeats could be from continued vaporization of Hg more deeply absorbed within the soil structure as opposed to from biotic activity. As such, changes induced by soil temperature rises can be as important as that of light exposure to the mechanism of Hg emissions from soil. This further suggests that Hg emissions flux from soils induced solely by either one of the two variables (temperature and light) may differ to a certain degree. This notwithstanding, both of these two abiotic variables are likely to interact effectively in the conversion of an increased percentage of oxidized Hg species into an elemental form resulting in subsequent emissions of the Hg from soil. Moreover, the results presented also make clear that temperature increases caused by climate changes will act to shift the equilibrium between mercury in air and mercury in soil towards higher concentrations in air. For real soils in higher latitudes, the expected range of average temperature might be in the range −10 to 30°C, whereas in more equatorial regions temperature might be expected to be in the range 15 to 55°C. Therefore, the results from this study apply to many of the surface temperatures found around the globe. However, we would expect that the results presented could be extrapolated to cover more extreme temperatures, as required.

## Figures and Tables

**Table 1:** Basic soil parameters and Hg content measured from 3 soil samples investigated in this study.

Parameter unit	pH	LOI^a^	CEC^b^	Hg
		(%)	*μ*eq g^−1^	Mg kg^−1^
S-1	7.01	6.03	142.9	3.309
S-2	6.34	4.36	42.6	2.942
S-3	6.47	5.68	79.3	2.787

^a^LOI: loss on ignition measured after 6 hrs at 450^°^C.

^b^CEC: cation exchange capacity.

**Table:** (a) Hg concentration exiting the chamber (ng m^−3^)

Temp	*C*(S1)^b^	*C*(S2)	*C*(S3-1)	*C*(S3-2)	*C*(S3-3)
25	7.065	3.046	6.568	NM	NM
30	10.412	2.607	4.206	DL	DL
40	21.194	6.159	3.941	DL	DL
50	32.524	4.160	1.822	DL	DL
60	13.024	6.565	1.386	0.064	1.025
70	NM	4.217	0.883	0.372	3.745

**Table:** (b) Hg emission flux (ng m^−2^ h^−1^)

Temp	*F*(S1)	*F*(S2)	*F*(S3-1)	*F*(S3-2)	*F*(S3-3)
25	70.2	30.3	65.3		
30	103.5	25.9	41.8		
40	210.6	61.2	39.2		
50	323.2	41.3	18.1		
60	129.4	65.2	13.8	0.6	10.2
70		41.9	8.8	3.7	37.2

**Table:** (c) Relationship between 1/*T* versus ln(emission flux)

1/*T* (abs)	ln(*F*(S1))	ln(*F*(S2))	ln(*F*(S3-1))	ln(*F*(S3-2))	ln(*F*(S3-3))
0.0034	4.2514	3.4100	4.1785		
0.0033	4.6392	3.2546	3.7329		
0.0032	5.3500	4.1142	3.6677		
0.0031	5.7782	3.7219	2.8961		
0.0030	4.8630	4.1780	2.6226	−0.4466	2.3207
0.0029		3.7354	2.1721	1.3071	3.6167

^a^Three soil samples are named as S1, S2, and S3, and the number of repetition is given after the hyphen.

^b^Initiation dates for each experiment: S1 (22 December 2009), S2 (30 December 2009), S3-1 (26 January 2010), S3-2 (2 February 2010), and S3-3 (11 February 2010).

**Table:** (a) Hg concentration exiting the chamber (ng m^−3^)

Temp	*C*(S1)^b^	*C*(S2)	*C*(S3-1)	*C*(S3-2)	*C*(S3-3)
25	17.101	3.684	7.502	NM	NM
30	14.702	3.116	4.782	0.089	0.016
40	48.746	7.647	9.515	0.328	0.131
50	60.455	12.350	34.146	1.316	0.699
60	38.728	10.452	56.805	4.208	2.559
70	NM	12.696	35.930	11.619	6.715

**Table:** (b) Hg emission flux (ng m^−2^ h^−1^)

Temp	*F*(S1)	*F*(S2)	*F*(S3-1)	*F*(S3-2)	*F*(S3-3)
25	169.9	36.6	74.5	NM	NM
30	146.1	31.0	47.5	0.9	0.2
40	484.4	76.0	94.6	3.3	1.3
50	600.7	122.7	339.3	13.1	6.9
60	384.8	103.9	564.5	41.8	25.4
70		126.2	357.0	115.5	66.7

**Table:** (c) Relationship between 1/*T* versus ln(emission flux)

1/*T* (abs)	ln(*F*(S1))	ln(*F*(S2))	ln(*F*(S3-1))	ln(*F*(S3-2))	ln(*F*(S3-3))
0.0034	5.1354	3.6003	4.3114		
0.0033	4.9842	3.4329	3.8612	−0.1230	−1.8362
0.0032	6.1829	4.3306	4.5492	1.1805	0.2617
0.0031	6.3981	4.8099	5.8269	2.5705	1.9384
0.0030	5.9528	4.6431	6.3359	3.7333	3.2360
0.0029		4.8376	5.8778	4.7488	4.2005

^a^Three soil samples are named S1, S2, and S3, and the number of repetition is given after the hyphen.

^b ^Initiation dates for each experiment: S1 (22 December 2009), S2 (30 December 2009), S3-1 (26 January 2010), S3-2 (2 February 2010), and S3-3 (11 February 2010).
